# Cloacael Carriage and Multidrug Resistance* Escherichia coli* O157:H7 from Poultry Farms, Eastern Ethiopia

**DOI:** 10.1155/2017/8264583

**Published:** 2017-02-27

**Authors:** Mude Shecho, Naod Thomas, Jelalu Kemal, Yimer Muktar

**Affiliations:** ^1^School of Veterinary Medicine, Wolaita Sodo University, Wolaita Sodo, Ethiopia; ^2^College of Veterinary Medicine, Haramaya University, P.O. Box 138, Dire Dawa, Ethiopia

## Abstract

A cross-sectional study was carried out to determine antimicrobial drug resistance patterns of* E. coli* O157:H7 isolates and estimate the level of the pathogen. A total of 194 cloacae swab samples were collected randomly in two poultry farms. Standard cultural, biochemical, and serological (latex agglutination) methods were used to isolate* E. coli* O157:H7. The isolates were subjected to antimicrobial susceptibility testing using disc diffusion method. Out of 194 cloacae samples examined, 13.4% (*n* = 26) were found to be positive for* E. coli* O157:H7. The finding indicated differences in* E. coli* O157:H7 infection among the different risk factors. Chicken from Adele Poultry Farm showed higher* E. coli* O157:H7 infection (OR = 3.89) than Haramaya University poultry farm and young birds had more infection (OR = 4.62) than adult birds. Of the total 14 antimicrobials included in the panel of study, the susceptibility results were varied with 96.15% and 0%* E. coli* O157:H7 isolates expressing resistance to erythromycin, clindamycin, spectinomycin, and ciprofloxacin, respectively. Multidrug resistance to more than two antimicrobial agents was detected in 24 (92.30%) of the isolates. The study showed high presence of antimicrobial resistant isolates of* E. coli* O157:H7. Further study is required to better understand the ecology and evolution of bacterial resistance to antimicrobial agents.

## 1. Introduction

Poultry is a major fast growing source of food in the world today [1]. However, it is one of the commodities most commonly associated with food-borne disease outbreaks. Pathogens can be transmitted to humans directly through contact with poultry litter or indirectly through contaminated poultry products [2]. The avian intestines have been considered as a reservoir of potential* E. coli *with zoonotic potential that could be transferred directly from birds to humans.

*E. coli *is a commensal bacterium in humans and animals and has a wide range of hosts. It is commonly present in the environment and considered an indicator of fecal contamination in food and water. It can acquire, maintain, and transmit resistance genes from other organisms in the environment.* E. coli* serotype O157:H7 is an enterohaemorrhagic strain, which was initially recognized in the United States of America, as a cause of food-borne illness, and has now emerged as an important enteric pathogen of considerable public health significance [3].

In animal production antimicrobials are widely used as growth promoter and in treatment of infectious diseases. The use of antimicrobials in poultry production industries for promotion of growth largely contributes to the high resistance to antimicrobial agents in normal flora of poultry and pathogenic microorganism [4]. The practice of using antimicrobials in feed may modify the intestinal flora by creating a selective pressure in favor of resistant bacteria populations (such as resistant* E. coli*) that could find their way into the environment and food chain [5]. Due to its ubiquity in humans and animals and its role as a pathogenic and commensal organism,* E. coli *has become one of the microorganisms that are commonly resistant to antimicrobials [6]. With the constant use of antimicrobials over a period of time, bacteria resist not only single but also multiple antimicrobials making some diseases troublesome to treat [7].

In recent years, antimicrobial resistance especially multidrug resistance has become very common in clinical isolates, including* E. coli *isolates of animal origin [8]. Antimicrobial resistance among* E. coli* in food animals such as chicken is of increasing concern due to the potential for transfer of these resistant pathogens to the human population [9]. Antimicrobial resistance is a global problem, and emerging antimicrobial resistance has become a public health fact worldwide [10]. The use of antimicrobials in food animals, as well as their role in promoting resistance in food-borne bacteria, is an important public health issue. To measure the baseline resistance rates and the impact of different targeted interventions, an ongoing monitoring system is necessary [6]. Even though there have been few studies about the level and antimicrobial resistance pattern of* E. coli *O157:H7 in poultry in Ethiopia, information is lacking. Thus, objectives of the study were to determine the antimicrobial resistance patterns of* E. coli *O157:H7 isolates and estimate the level of the pathogen in apparently healthy birds.

## 2. Materials and Methods 

### 2.1. Study Site Description and Study Population

**Figure 1:**
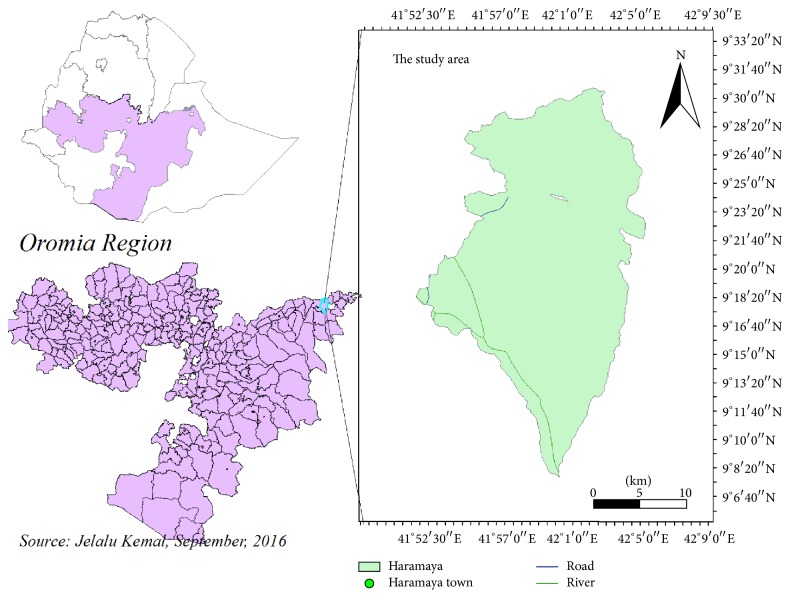
Map showing the study area.

The study was conducted at Haramaya district Adele Poultry Farm and poultry farm in Haramaya University. Haramaya district is located in eastern Hararge Zone of Oromia Regional State, along the high way from Addis Ababa to Harar 508 km from Addis Ababa and 19 km ahead to reach Harar at an altitude of 1980 meters above sea level (m.a.s.l.), 9°26′N latitude and 42°3′E longitude (Figure 1). The mean annual rainfall is 780 mm. The mean annual minimum and maximum temperatures are 8.5 and 24.4°C, respectively. Haramaya University poultry farm is located at 42°3′E longitude, 9°26′N latitude, and an elevation of 1980 m.a.s.l. and 513 km away from Addis Ababa. The annual mean rain fall of the area amounts to 780 mm and the average minimum and maximum temperature are 8°C and 24°C, respectively. The total poultry population of the country is estimated to be 56.87 million and it comprises 95.86% indigenous breeds, 2.79% crossbreeds, and 1.35% exotic breeds [12].

This study was conducted on exotic breed chicken under intensive management system. The target population was apparently healthy exotic breed of white leg horn and Feyumi (Egyptian breed) breed chickens. Both farms comprise the aforementioned breeds. Most of these breeds have been from Debre Zeit farms and the fertilized egg was imported from Egypt, Holland, and other countries. Poultry were selected according to their age groups and breeds. The age was conveniently subdivided into young growers up to six months of age and adult chicken. The main purpose of these poultry farms is to supply egg, live chicken for meat, and 3-month-old chick to the surrounding farmers and backyard and private producers. Furthermore, Haramaya University satisfies its egg demand for its cafeteria of all campuses and the community residing within the university from own farm production. The farms use formulated feed either buying from Debre Zeit or formulating feed on their farm by mixing with local available cereals, pulses, university cafeteria and staff lounge leftovers, and carcass from abattoirs in order to reduce the cost of input.

### 2.2. Study Design

A cross-sectional purposive type of study was conducted from October 2015 to May 2016 aimed to isolate, identify, and determine the antimicrobial susceptibility profiles* E. coli* O157:H7 in the area. A total of 194 samples of cloacae swabs were collected randomly from healthy chickens from two poultry farms located in Haramaya University (*n* = 106) and Haramaya district Adele Poultry Farm (*n* = 88), eastern Ethiopia. During the study hypothesized risk factors such as the age, breed, and farm location of the birds were taken into account and recorded.

### 2.3. Sample Collection

All samples were taken using sterile swabs which were moistened with sterile buffered peptone water (Oxoid Ltd., Cambridge, UK), placed in sterile vial tubes containing 8 mL buffered peptone water which is used to avoid drying out of the swabs. Samples were kept in ice box containing ice pack for transporting to Haramaya University, College of Veterinary Medicine, Microbiology Laboratory, immediately for further analysis.

### 2.4. Isolation and Identification of* E. coli* O157:H7

Isolation and identification of* E. coli *O157:H7 were performed by standard bacteriological methods. The samples were incubated at 37°C for 24 hrs on the same day upon arrival at the laboratory on MacConkey agar (Oxoid Ltd., Cambridge, UK) which is selective and differential medium for* E. coli* [13]. A pink colony was picked and subcultured on Eosin Methylene Blue (EMB) agar (Oxoid Ltd., Cambridge, UK) to obtain pure colony. Colonies with metallic green sheen on EMB (characteristic of* E. coli*) were later characterized microscopically using Gram's stain according to the method described by Merchant and Packer [14]. After isolation of the organism on the selective media, differential screening media, triple sugar iron (TSI) agar (Difco, MI, USA) was used for further characterization. Yellow slant, yellow butt, presence of gas bubbles, and absence of black precipitate in the butt was observed which indicates* E. coli* [15]. Then the isolates were subjected to different biochemical tests according to Quinn et al. [16] such as sugar fermentation test and indole production test, methyl-red, Voges-Proskauer, and citrate utilization (IMViC) test. Then the bacterium that was confirmed as* E. coli* was subcultured onto Sorbitol MacConkey agar (Oxoid Ltd., Cambridge, UK) from nutrient agar and colorless colonies (nonsorbitol fermenter) were again subcultured onto nutrient agar and latex* E. coli* O157:H7 agglutination test was performed to determine strains using polyvalent antisera (DENKA SEIKEN Co. Ltd., Tokyo, Japan).

### 2.5. Antimicrobial Susceptibility Testing for* E. coli* O157:H7 Isolates

The antimicrobial susceptibility testing* E. coli* O157:H7 isolates was conducted using disc diffusion method (Kirby-Bauer method) on Mueller-Hinton agar (Oxoid Ltd., Cambridge, UK) according to the guidelines of the Clinical and Laboratory Standards Institute [11]. All* E. coli* O157:H7 isolates were evaluated for antimicrobial susceptibility to 14 antimicrobial agents. A McFarland 0.5 (the turbidity of the test broth was adjusted with saline until the turbidity of the test suspension equated that of the standard) standardized suspension of the bacteria in tryptone soya broth (Oxoid Ltd., Cambridge, UK) was prepared. A bacterial suspension incubated for 6–8 hours was swabbed over the entire surface of Mueller-Hinton agar (Oxoid Ltd., Cambridge, UK) with a sterile cotton swab. The inoculated pates were allowed to stand for 3–5 minutes to observe any excess moisture from the medium before the antimicrobial discs were applied. A ring of discs containing single concentrations of each antimicrobial agent was then placed onto the inoculated surface using disc dispenser, gently pressed with the point of the forceps for ensuring complete contact with the agar surface, and then inverted. After 16–18 hours of incubation at 35°C ± 2°C, aerobically, clear zones produced by antimicrobial inhibition of bacterial growth were measured in mm using a measuring caliper. For the susceptibility testing, the following 14 antimicrobial drugs and concentrations were used: ampicillin (10 *μ*g), amoxicillin (20 *μ*g), cefoxitin (30 *μ*g), chloramphenicol (30 *μ*g), ciprofloxacin (5 *μ*g), clindamycin (30 *μ*g), erythromycin (15 *μ*g), gentamycin (10 *μ*g), kanamycin (30 *μ*g), nalidixic acid (30 *μ*g), spectinomycin (30 *μ*g), streptomycin (10 *μ*g), tetracycline (30 *μ*g), and trimethoprim (5 *μ*g) (Oxoid Ltd., Cambridge, UK). The antimicrobials used were selected from the currently available and commonly used chemotherapeutic agents for the treatment of* E. coli* infection in humans and animals.* E. coli* ATCC25922 and* E. coli* ATCC35218 were used as quality control during the test. Finally, the findings of antimicrobial resistance testing were recorded as susceptible, intermediate, and resistant according to Clinical and Laboratory Standards Institute break points [11] (Table 1).

### 2.6. Data Analysis

All the data were coded and entered into Microsoft Excel® 2007. The data were then exported to SPSS windows version 20.0 (SPSS) (IBM, Armonk, USA) for appropriate statistical analysis. The occurrence of the pathogen was determined by using descriptive statistics. Chi square (*χ*^2^) and odds ratio were used to measure the association between the different risk factors and occurrence of* E. coli* O157:H7 in chicken cloacae. Effects were reported as statistically significant if *P* value is less than 5% (*P* < 0.05).

## 3. Results

### 3.1. Level of* E. coli* O157:H7 from Cloacal Fecal Sample

Based on colonial morphology and biochemical and latex agglutination tests,* E. coli* O157:H7 were isolated from cloacal swab sample of chickens (Table 2). Out of the 194 cloacae samples examined, 26 (13.4%) were found positive for* E. coli* O157:H7. The results indicated different level of* E. coli* O157:H7 among the different selected risk factors (source, age, and breed) of examined poultry; Haramaya University poultry farm (7; 6.6%), Adele Poultry Farm (19; 21.6%), young (21; 18.8%), adult chicken (5; 7.5%), White Leghorn (19; 15.6%), and Feyumi (7; 10.6%) showed level of* E. coli* O157:H7, respectively. There was a significant difference in* E. coli* O157:H7 among the farms and age groups (*P* < 0.05). Chicken from Adele Poultry Farm showed* E. coli* O157:H7 infection four times higher than Haramaya University poultry farm and young birds had more infection than adult birds. However there was an equal chance of* E. coli* O157:H7 infection among different breeds of chicken.

A total of 26 isolates of* E. coli* O157:H7 were analyzed, 7 from Haramaya University poultry farm and 19 from Adele Poultry Farm for antimicrobial resistance test. The percentage of isolates susceptible, intermediate, and resistant to each antimicrobial agent is outlined in Table 3. Of the total 14 antimicrobials included in the panel of study, the susceptibility results were varied with 96.15% and 0%* E. coli* O157:H7 isolates expressing resistance to erythromycin, clindamycin, spectinomycin, and ciprofloxacin, respectively. The isolates expressed resistance to ampicillin and amoxicillin at frequencies of 92.30% and 34.61%, respectively. Cefoxitin and tetracycline resistance occurred at a frequency of 84.61% and 76.92%, respectively. Relatively similar resistance was observed among kanamycin (15.38), nalidixic acid (23.07), and streptomycin (34.61) while lower resistance was recorded between chloramphenicol (3.84) and gentamycin (7.69). Ciprofloxacin, chloramphenicol, trimethoprim, gentamicin, and streptomycin were the most sensitive antibiotics in the study. Intermediate resistance/susceptibility to various antibiotics were observed for 0–46.15%* E. coli* O175:H7 strains.

The level of multiple resistance patterns in* E. coli* O157:H7 isolates is given in Table 4. Single and multiple resistance to most of the antimicrobials tested were observed. Multidrug resistance was recorded in case of 2–9 antimicrobials for the tested strains. Multidrug resistance to more than two antimicrobial agents was detected in 24 (92.30%) of the isolates. One isolate was resistant to up to nine antimicrobials tested. The resistance pattern most frequently observed in the isolates was resistance to erythromycin in combination with clindamycin, ampicillin, and cefoxitin 3 (12.5%). The next most frequent resistance isolates were resistance to erythromycin, cefoxitin, clindamycin, ampicillin, amoxicillin, tetracycline, and kanamycin 2 (8.33%). Multidrug resistance was defined as resistance exhibited to two or more antimicrobials.

**Figure 2:**
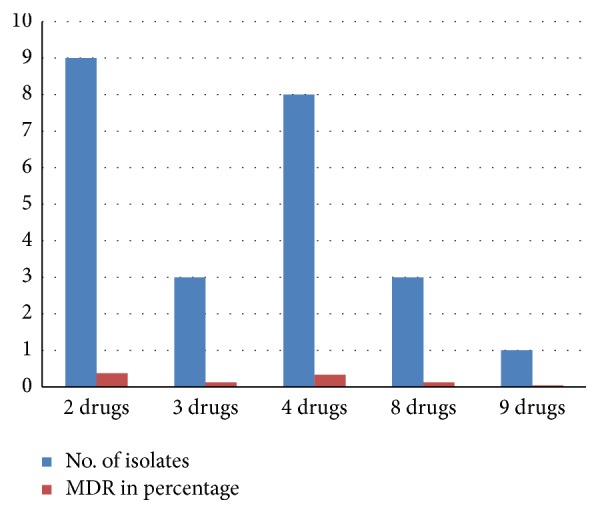
Multidrug resistant profiles of* E. coli* O157:H7 isolates of the farms.

Among the* E. coli* O157:H7 isolates, 37.5%, 33.33%, and 4.16% expressed resistance to two, four, and nine antimicrobials, respectively (Table 4; Figure 2), and resistance to three and eight antimicrobials occurred at a frequency of 12.5% each. 8.33% of the isolates showed resistance to a single antimicrobial (kanamycin, tetracycline, ampicillin, and gentamycin).

## 4. Discussion 

The occurrence of* E. coli* O157:H7 among poultry farms varies considerably [17]. Several studies showed 0.0% to 27.8% level of* E. coli* O157:H7 on poultry farms in different countries [18–20]. In the current study, 13.4% (*n* = 26) of* E. coli* O157:H7 was isolated from cloacal samples taken from poultry farms which agrees with the findings of Olatoye et al.  [21] who reported 13 and 14% level of* E. coli* O157:H7 from Lagos and Ibadan poultry farms, respectively. In another study, Ojo et al.  [22] confirmed* E. coli *O157:H7 strains in the faeces of poultry sampled from different farms in Nigeria and Aibinu et al.  [23] also isolated* E. coli *O157:H7 from chicken in Lagos and Ogun State in Nigeria who found 14.5%.

Moderately comparable levels of* E. coli* O157:H7 were reported from different countries: 8% [24] and 8.1% [25] in Ethiopia, 9% [26] in India, and 6% [27] in Turkey. However, the current finding is higher than the reports of Baran and Gulmez [28], Dutta et al. [29], and McCluskey et al. [30] who reported 2%, 3.2%, and 2.8% level of* E. coli* O157:H7 in Canada, the United Kingdom, and South Africa, respectively. In addition, 4.4% occurrence was reported in Kenya [31]. These variations might be due to different sampling techniques, areas, and time and lack of strict hygienic measures among the farms and cross contamination with other principal reservoirs [24] and also due to the low isolation rate of culture methods compared to more sensitive immunological and molecular methods [32].

Chicken from Adele Poultry Farm showed* E. coli* O157:H7 infection four times higher than Haramaya University poultry farm and young birds had more infection than adult birds. However there was an equal chance of* E. coli* O157:H7 infection among different breeds of chicken. Zhao et al. [33] described young animals tend to carry* E. coli* O157:H7 more frequently than adults. Moreover, as young chicks are not fully immunocompetent and have also lost protection from maternal antibodies [34]. Regarding* E. coli* O157:H7 infection variation between the two farms might be due to security differences among the farms. However there was no statistically significant association between different breeds; this might be due to equal chance of infection among the breeds.

Antimicrobial resistance has become a global concern [35]. Indiscriminate use of antimicrobial agents in humans, veterinary, and agriculture is considered the most important factor promoting the emergence, selection, and dissemination of antimicrobial resistant microorganisms in both veterinary and human medicine [36]. There were variations in antimicrobial susceptibility of* E. coli* O157:H7 isolates in the present study. A complete (100%) susceptibility was observed against ciprofloxacin and large numbers of isolates were also found to be susceptible against chloramphenicol (96.15%), trimethoprim (92.30), gentamycin (88.46%), spectinomycin (69.23%), streptomycin (65.38%), and nalidixic acid (61.53%). Zinnah et al. [37] reported high susceptible* E. coli* isolates against ciprofloxacin. Similar to the current finding Hailu and Tefera [38] had reported susceptible* E. coli* O157:H7 isolates to chloramphenicol (100%), spectinomycin (62.61%), and nalidixic acid (61.76%). Taye et al. [39] also reported most isolated strains were found susceptible to chloramphenicol and spectinomycin. Closely related chloramphenicol susceptibility to our finding was also reported by Hamisi et al. [13] and Talebiyan et al. [40] from Tanzania and Iran, respectively. The current study finding is distantly related to the finding of Moniri and Dastehgoli [41] who found 36% of healthy broilers susceptibility to chloramphenicol and with the finding of Zakeri and Kashefi [42] who reported 51% from cases of colibacillosis in Iran. 100% chloramphenicol resistance isolates were reported by Islam et al. [43] from poultry in Dhaka, Bangladesh, which is in disagreement with the current study finding.

Comparable susceptible isolates (94.34%) were reported by Talebiyan et al. [40] from chickens in Iran. Muhammad et al. [44] reported 80% susceptible* E. coli* isolate to gentamicin from Bangladesh which is comparable to our finding. Gentamicin was also observed in 71.7%* E. coli* O157:H7 isolates of poultry sample in Saudi Arabia [45]. In another work, Miles et al. [35] had reported gentamicin susceptibility to all tested* E. coli* isolates. According to the report of Hassan [46] isolates were also 100% susceptible for gentamicin in layer poultry reported from Bangladesh. Susceptibility to gentamicin in the current study is distantly related to finding of Zinnah et al. [37] who reported 40% in Bangladesh. Resistance gentamicin was reported to 46.6% isolates by Abd El Tawab et al. [47] from broiler chickens which is higher than the current study. In contrast to the present finding low susceptible* E. coli* isolates were reported to nalidixic acid (29.7%) [45], spectinomycin, trimethoprim (6%) [48], and streptomycin (30%) [44].

Antimicrobial resistance to clindamycin (96.15%), erythromycin (96.15%), ampicillin (92.30%), cefoxitin (84.61%), and tetracycline (76.92%) was noted in* E. coli* O157:H7 isolates (Table 3). The presence and frequency of drug resistance in* E. coli* O157:H7 from cloacal samples agree with findings of other studies on antimicrobial resistance in* E. coli* [38, 49, 50]. High level of* E. coli* isolates resistant to ampicillin, erythromycin, and amoxicillin were also reported by former study [37]. Similar resistant isolates to tetracycline were reported by different researchers [4, 47, 51, 52]. Resistance patterns of these drugs could be due to the widespread, indiscriminate, and lengthy use of the drugs in the poultry farms [49, 53]. Bacteria can be exposed to these antimicrobial agents in nature and used for disease treatment, for prophylaxis, or for livestock growth promotion which can lead to resistance. Plasmid mediated with a wide variety of genetic determinants also contributes to resistance in these antimicrobials [54]. This makes it more possible for a susceptible bacterium to acquire resistance factors through conjugation or transformation [35]. Furthermore, the problem is probably associated with the widespread use of these antimicrobials in humans and animals for treatment of enteric infections.

In the present study, multidrug resistance to more than two antimicrobial agents was detected in 24 (92.30%) of the isolates. The resistance pattern most frequently observed in the isolates was resistance to erythromycin in combination with clindamycin, ampicillin, and cefoxitin 3 (12.5%). The next most frequent resistance isolates were resistance to erythromycin, cefoxitin, clindamycin, ampicillin, amoxicillin, tetracycline, and kanamycin 2 (8.33%) (Table 4). Among the* E. coli* O157:H7 isolates, 37.5%, 33.33%, and 4.16% expressed resistance to two, four, and nine antimicrobials, respectively (Table 4; Figure 2), and resistance to three and eight antimicrobials occurred at a frequency of 12.5% each. 8.33% of the isolates showed resistance to a single antimicrobial (Figure 2).

Similar findings on multidrug resistance of* E. coli *strains has been reported from Ethiopia [38, 39, 51] and other parts of the world (Khan et al. 2002; [33, 37, 43, 44, 55, 56]). Such high incidence of multidrug resistance may apparently have occurred due to indiscriminate utilization of antimicrobial agents which may ultimately replace the susceptible microorganisms [53, 57]. Feed additives for poultry suggest encountering such resistance emergence with reduced and unsafe application of antimicrobials in animal farming and clinical purposes. The multidrug resistance observed in this study might also be mediated by genetic mobile elements such as plasmids, transposons, and integrons as seen in the case of other studies.

## 5. Conclusion

The study showed 13.4% of* E. coli *O157:H7 in cloacal swab samples in the study poultry farms. Chickens younger than six months had significantly higher level of* E. coli* O157:H7 compared to older chickens. A significant variation of infection was also recorded in Adele Poultry Farm compared to Haramaya University poultry farm. The study showed the presence of antimicrobial resistant isolates of* E. coli *O157:H7 in the studied poultry farms.* E. coli *O157:H7 isolates showed high level resistance to clindamycin, erythromycin, ampicillin, cefoxitin, and tetracycline which are commonly used antimicrobial agents in veterinary and human practices. The vast majority of* E. coli* O157:H7 isolates showed multiple drugs resistance for two to nine antimicrobials. This could have a significant public health consequence if these microorganisms are transmitted to humans through food chain. Therefore, further study is required to better understand bacterial resistance to antimicrobial agents with emphasis on surveillance of multidrug resistant* E. coli* O157:H7 isolates.

## Figures and Tables

**Table 1:** Zone diameter interpretive standard chart for *Enterobacteriaceae* [11].

Antimicrobial agents and symbols	Disc potency (*µ*g)	Zone diameter, nearest whole mm		
		Resistance	Intermediate	Susceptible
Amoxicillin (AML)	20	≤13	14–16	≥17
Ampicillin (AMP)	10	≤13	14–16	≥17
Cefoxitin (FOX)	30	≤14	15–17	≥18
Chloramphenicol (C)	30	≤12	13–17	≥18
Ciprofloxacin (CIP)	5	≤14	15–17	≥21
Clindamycin (CLI)	30	≤16	—	≥17
Erythromycin (E)	15	≤13	14–22	≥18
Gentamycin (CN)	10	≤12	13-14	≥15
Kanamycin (K)	30	≤13	14–17	≥18
Nalidixic acid (NAL)	30	≤17	—	≥18
Spectinomycin (SPT)	30	≤11	12–14	≥15
Streptomycin (S)	10	≤11	12–14	≥15
Tetracycline (TE)	30	≤11	12–14	≥15
Trimethoprim (TRI)	5	≤13	14–17	≥17

**Table 2:** Level of *E. coli* O157:H7 isolates with different hypothesized risk factors from Haramaya University and Adele Poultry Farms.

Risk factors	Risk categories	Number examined	Number positive	Proportion (%)	OR (95% CI)	*P* value
Source	HUPF^a^	106	7	6.6	1	
	APF^b^	88	19	21.6	3.89 (1.5–11.5)	0.002
Age	Adult	93	5	7.5	1	
	Young	101	21	18.8	4.62 (1.6–16.3)	0.021
Breed	Feyumi	85	7	10.6	1	
	White Leghorn	109	19	15.6	2.35 (0.9–6.9)	0.310

*Total*		*194*	*26*	*13.4*		

^a^Haramaya University Poultry Farm.

^b^Adele Poultry Farm.

**Table 3:** Antimicrobial resistance profiles of isolated *E. coli *O157:H7 from Haramaya University and Adele Poultry Farms.

Antimicrobial agents	Disc potency (*µ*g)	Number of isolates	Susceptible *N* (%)	Intermediate *N* (%)	Resistant *N* (%)
Amoxicillin	20	26	7 (26.92)	10 (38.46)	9 (34.61)
Ampicillin	10	26	0 (0.0)	2 (7.69)	24 (92.30)
Cefoxitin	30	26	2 (7.69)	2 (7.69)	22 (84.61)
Chloramphenicol	30	26	25 (96.15)	0 (0.0)	1 (3.84)
Ciprofloxacin	5	26	27 (100)	0 (0.0)	0 (0.0)
Clindamycin	30	26	0 (0.0)	1 (3.84)	25 (96.15)
Erythromycin	15	26	1 (3.84)	0 (0.0)	25 (96.15)
Gentamycin	10	26	23 (88.46)	1 (3.84)	2 (7.69)
Kanamycin	30	26	10 (38.46)	12 (46.15)	4 (15.38)
Nalidixic acid	30	26	16 (61.53)	3 (11.53)	6 (23.07)
Spectinomycin	30	26	18 (69.23)	8 (30.76)	0 (0.0)
Streptomycin	10	26	17 (65.38)	0 (0.0)	9 (34.61)
Tetracycline	30	26	2 (7.69)	4 (15.38)	20 (76.92)
Trimethoprim	5	26	24 (92.30)	0 (0.0)	2 (7.69)

**Table 4:** Resistance patterns of *E. coli* O157:H7 isolates form Haramaya University and Adele Poultry Farms against 14 antimicrobial agents.

Antimicrobials	*E. coli* O157:H7	
	Frequency	%
E, K	1	4.16
E, TE	2	8.33
E, C	1	4.16
E, CLI	1	4.16
E, SPT	1	4.16
E, AMP	2	8.33
E, FOX	1	4.16
E, CLI, TE	1	4.16
E, K, TE	1	4.16
CN, E, TE	1	4.16
E, C, CLI, AMP	3	12.5
E, FOX, C, AML	1	4.16
E, CLI, C, AMP	1	4.16
E, S, C, AML	1	4.16
E, AMP, TE, AML	1	4.16
CN, E, S, AMP	1	4.16
E, CLI, K, AMP, FOX, C, TE, AML	2	8.33
E, CLI, K, AMP, C, TE, TRI, AML	1	4.16
E, S, K, NAL, CLI, C, TE, TRI, AML	1	4.16

*Total*	*24*	*92.30*

E: erythromycin, S: streptomycin, Nal = nalidixic acid, K: kanamycin, AMP: ampicillin, TE: tetracycline, RIT: trimethoprim, AML: amoxicillin, CIP: ciprofloxacin, CN: gentamycin, FOX: cefoxitin, C: chloramphenicol, and SPT: spectinomycin.
